# Reduced mean platelet volume (MPV) as an inflammatory marker in Chinese women with polycystic ovary syndrome: a case-control study

**DOI:** 10.1186/s12905-025-04018-1

**Published:** 2025-10-10

**Authors:** Qianqian Yin, Ying Liu, Guoqing Zhang, Yijuan Cao, Jianhua Zheng

**Affiliations:** 1 Center for Reproductive Medicine, XuZhou Central Hospital, Southeast University Affiliated Xuzhou Central Hospital, No. 199, Jiefang Road, Xuzhou, Jiangsu 221009 China; 2 Jiangsu Key Laboratory of New Drug Research and Clinical Pharmacy, Xuzhou Medical University, No. 209, Tongshan Road, Xuzhou, Jiangsu 221004 China; 3 Department of Obstetrics and Gynecology, XuZhou Central Hospital, Southeast University Affiliated Xuzhou Central Hospital, No. 199, Jiefang Road, Xuzhou, Jiangsu 221009 China

**Keywords:** Mean platelet volume, Platelet, Neutrophil, Lymphocyte, Polycystic ovary syndrome

## Abstract

**Background:**

Low-grade chronic inflammation is observed in women with polycystic ovary syndrome (PCOS). A recent genome-wide association study (GWAS) suggested that several inflammation marker levels were altered in the blood routine tests in PCOS patients and such changes were associated with variant gene clusters. Therefore, inflammatory marker alterations in the blood routine tests are significant and might be involved in the onset as well as progression of PCOS. Compared with other markers, these inflammatory markers in the blood routine tests are less expensive and easier to detect. Very few studies have evaluated the changes in platelet indicators like platelet count and mean platelet volume (MPV) in Chinese women with PCOS. The aim of the study was to investigate the changes in platelet-related inflammatory indices in Chinese women with PCOS.

**Methods:**

The study included 299 women aged 18–47 with PCOS and 481 healthy women as the control group. We conducted propensity score matching to refine our dataset, utilizing the ‘Matching’ package in R to match cases with the disease to controls. Correlation analyses were performed using Pearson correlation analysis. Partial correlation analysis was used to analyse the correlation between MPV and the indicators after adjusting for age, body mass index (BMI) and waist circumference (WC). Binary logistic regression (forward-wald) was used to evaluate if the relationship between MPV and PCOS was independent of obesity, insulin resistance (IR), and hyperandrogenism.

**Results:**

After matching the age, BMI, and WC of the two groups, high-sensitivity CRP (hs-CRP), white blood cell counts (WBCs), lymphocytes, platelets and great platelet count (GPC) were significantly higher (*P* = 0.007, 0.022, 0.011, 0.014, and 0.000, respectively), and mean platelet volume (MPV) and platelet distribution width (PDW) were significantly lower in PCOS patients compared with the control group (*P* = 0.000, 0.016, respectively). Neutrophils, neutrophil-to-lymphocyte ratio (NLR), and platelet hematocrit (PCT) did not show statistically significant differences between the two groups (*P* = 0.196, 0.480, 0.646, respectively). Decreased MPV was independently associated with the occurrence of PCOS (*P* = 0.005, OR = 0.770, 95% CI, 0.641–0.925). MPV was negatively correlated with total testosterone (TT), free testosterone (FT) and total cholesterol (CHOL) levels after correcting for age and obesity (*r* = -0.149, *P* = 0.000; *r* = -0.093, *P* = 0.010; *r* = -0.081, *P* = 0.025; respectively).

**Conclusions:**

Chinese PCOS patients have significantly reduced MPV as an inflammatory indicator independent of obesity, IR, and dyslipidemia.

## Introduction

Polycystic ovary syndrome (PCOS), a prevalent reproductive endocrine disorder, affects 6–20% of women of reproductive age globally, with variation depending on diagnostic criteria [1, 2]. Moreover, PCOS is also associated with several other long-term adverse health outcomes like obesity, dyslipidemia, insulin resistance (IR), type 2 diabetes mellitus, and coronary artery disease [2, 3].

Although the pathophysiology of PCOS remains incompletely understood, genetic factors clearly contribute to its development [4]. Stamou et al. recently employed genome-wide association studies (GWAS) to cluster 60 PCOS-associated genetic variants and 49 phenotypic traits, revealing four distinct genetic clusters linked to PCOS phenotypes [2]. They also observed altered levels of several blood inflammatory markers in PCOS patients, including leukocytes, lymphocytes, and mean platelet volume (MPV), which correlated with specific gene clusters. Therefore, these inflammatory marker alterations in the blood routine tests are significant and might be involved in the onset as well as progression of PCOS.

Routine blood tests offer a cost-effective and accessible method for measuring inflammatory markers compared to other biomarkers. Previously, leukocytes, neutrophils, lymphocytes, and the neutrophil-to-lymphocyte ratio (NLR) were the primary focus of PCOS patients’ routine blood indices. Very few studies have evaluated the changes in platelet indicators like platelet count and MPV. Moreover, existing research on platelet indices in PCOS derives mainly from Turkish and Saudi Arabian populations [5], despite known racial variations in these hematological parameters [6, 7]. This study therefore examines platelet-related inflammatory markers specifically in Chinese women with PCOS.

## Materials and methods

### Subjects and study design

This study was conducted in the Department of Obstetrics and Gynecology and Reproductive Clinic at Xuzhou Central Hospital from July 2019 to September 2024. It was approved by the Xuzhou Central Hospital’s institutional review board. Written informed consent was obtained from all participants.

According to our group’s pre-published study, patients were diagnosed with PCOS based on the Rotterdam criteria [8]. Controls were defined as patients with regular menstrual cycles without hyperandrogenism or polycystic ovarian changes. However, patients with any recent acute or chronic illness (including recent history of infection, endometriosis, malignancy, autoimmune diseases, haematological diseases, etc.) as well as those on medications that could affect our results, were excluded.

We included 299 PCOS female patients and 481 controls. All participants were aged between 18 and 47 years.

### Study protocol

Fasting blood samples were obtained from all PCOS patients between the first and fifth day of menstrual period/withdrawal bleeding. Subsequently, prolactin, total testosterone (TT), free testosterone (FT), and thyroid-stimulating hormone (TSH) were assessed by chemiluminescence immunometric assay (Beckman Unicel DxI 800; Snibe MAGLUMI 4000; Abbott Immulite 2000 analyzer). Moreover, fasting plasma glucose (FPG) and fasting insulin (FIN) levels were measured using the glucose oxidase method (Hitachi 7600 autoanalyzer) and chemiluminescence immunometric assay (Roche e601 analyzer), respectively. Additionally, total cholesterol (CHOL), triglycerides (TG), high-density lipoprotein cholesterol (HDL-c), and low-density lipoprotein cholesterol (LDL-c) were measured using an enzymatic colorimetric method (Hitachi 7600 autoanalyzer). An automatic blood cell counter (SYSMEX 2800R analyzer) was used to process 2 mL of venous blood samples drawn into ethylenediaminetetraacetic acid tubes and processed within 2 h post-venipuncture. Homeostasis model assessment-insulin resistance (HOMA-IR) was calculated as: HOMA-IR = (FIN [mU/L] × FPG [mmol/L])/22.5.

#### Statistical analysis

Data were analyzed using SPSS 24.0 statistical software. We preliminarily conducted a sample size estimation. Initially, we collected samples from 54 cases, including 29 patients with PCOS and 25 cases in the control group. The MPV levels in PCOS patients were 10.16 ± 1.08, while in the control group, they were 10.55 ± 1.07. Under the conditions of α = 0.05 and 1-β = 0.90, our sample size estimation indicated that 161 cases per group would be required. All continuous variables’ data were expressed as mean ± standard deviation (mean ± SD). We conducted propensity score matching to refine our dataset, utilizing the ‘Matching’ package in R to match cases with the disease to controls. Correlation analyses were performed using Pearson correlation analysis. Partial correlation analysis was used to analyse the correlation between MPV and the indicators after adjusting for age, body mass index (BMI) and waist circumference (WC). Binary logistic regression (forward-wald) was used to evaluate if the relationship between MPV and PCOS was independent of obesity, IR, and hyperandrogenism. A statistically significant difference was defined as *p* ≤ 0.05.

## Results

### Comparison between women with and without PCOS

**Fig. 1:**
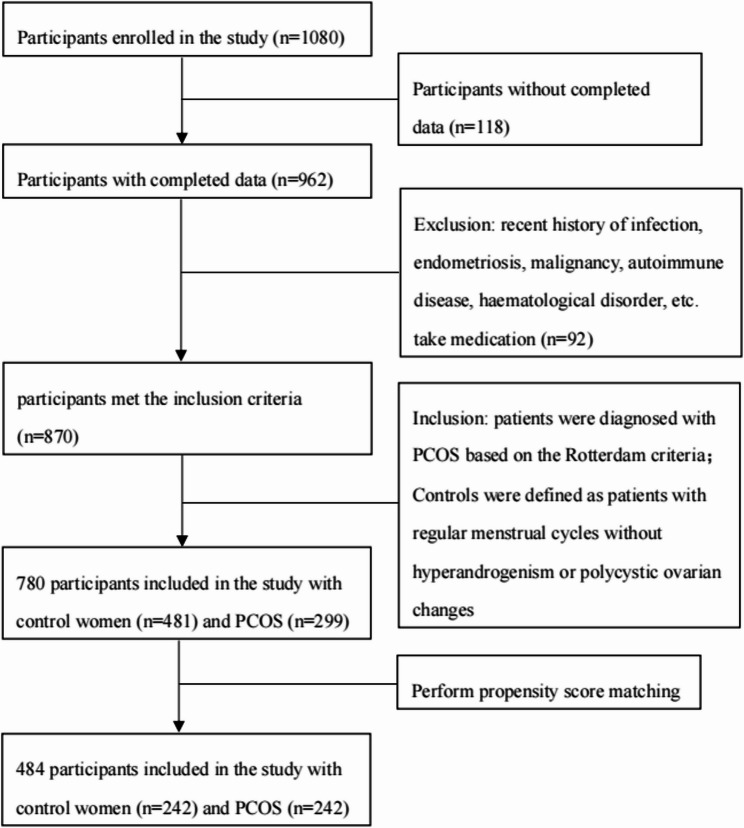
Flow chart of participant recruitment

Figure 1 illustrates the participant enrollment process. Table 1 presents the baseline characteristics of the study population. The PCOS patients were younger, as well as had greater BMI and WC than those without PCOS (*p* = 0.000, 0.000, 0.000, respectively). Women with PCOS exhibited elevated androgen levels and significant metabolic disturbances, including dysregulated blood glucose, insulin, and lipid profiles, relative to controls. (TT, *p* = 0.000; FT, *p* = 0.000; FPG, *p* = 0.001; FIN, *p* = 0.000; HOMA-IR, *p* = 0.000; CHOL, *p* = 0.023; TG, *p* = 0.000; HDL-c, *p* = 0.000; and LDL-c, *p* = 0.000; respectively). Women with PCOS exhibited elevated levels of high-sensitivity CRP (hs-CRP), white blood cells (WBCs), neutrophils, lymphocytes, platelets, platelet hematocrit (PCT), and large platelet count ratio (GPC), alongside decreased MPV and platelet distribution width (PDW) relative to controls (*p* = 0.000, 0.001, 0.001, 0.000, 0.001, 0.000, 0.000, and 0.016, respectively). The NLR values did not differ significantly between the two groups (*p* = 0.589).

**Table 1:** Clinical and laboratory characteristics of whole cohort of polycystic ovary syndrome (PCOS) subjects (*n* = 481) and controls (*n* = 299) and a subgroup of women with (*n* = 242) and without (*n* = 242) PCOS matched for age and obesity

	Whole cohort			age, BMI, WC-matched cohort		
	Control (*n* = 481)	PCOS (*n* = 299)	*P*	Control (*n* = 242)	PCOS (*n* = 242)	*P*
Age (years)	32.58 ± 4.56	30.16 ± 4.09	0.000	30.86 ± 3.89	30.72 ± 3.92	0.692
WC (cm)	80.21 ± 8.87	85.76 ± 10.52	0.000	82.97 ± 9.16	83.50 ± 9.60	0.532
BMI (kg/m^2^)	23.03 ± 3.23	25.53 ± 4.07	0.000	24.34 ± 3.42	24.50 ± 3.66	0.619
TT (ng/dL)	42.25 ± 14.85	63.91 ± 22.38	0.000	43.74 ± 15.36	63.05 ± 22.15	0.000
FT (pg/mL)	1.67 ± 0.75	2.46 ± 1.13	0.000	1.76 ± 0.80	2.44 ± 1.13	0.000
FPG (mmol/L)	5.49 ± 0.55	5.70 ± 0.96	0.001	5.56 ± 0.62	5.60 ± 0.72	0.427
FIN (mU/L)	11.78 ± 6.36	18.27 ± 12.98	0.000	13.55 ± 7.20	15.56 ± 9.49	0.009
HOMA-IR	2.92 ± 1.73	4.87 ± 4.45	0.000	3.41 ± 1.98	3.99 ± 2.75	0.008
CHOL (mmol/L)	4.56 ± 0.78	4.69 ± 0.85	0.023	4.60 ± 0.74	4.72 ± 0.87	0.106
TG (mmol/L)	1.10 ± 0.73	1.43 ± 0.84	0.000	1.21 ± 0.77	1.36 ± 0.82	0.032
HDL-c (mmol/L)	1.41 ± 0.29	1.28 ± 0.27	0.000	1.38 ± 0.27	1.33 ± 0.27	0.034
LDL-c (mmol/L)	2.58 ± 0.67	2.81 ± 0.76	0.000	2.67 ± 0.67	2.82 ± 0.78	0.017
hs-CRP (mg/L)	1.01 ± 1.49	1.88 ± 2.22	0.000	1.19 ± 1.56	1.60 ± 1.79	0.007
WBC (10^9^/L)	6.01 ± 1.53	6.59 ± 1.52	0.000	6.13 ± 1.52	6.45 ± 1.50	0.022
Neutrophils (10^9^/L)	3.72 ± 1.28	4.04 ± 1.22	0.001	3.84 ± 1.27	3.99 ± 1.23	0.196
Lymphocytes (10^9^/L)	1.82 ± 0.52	2.04 ± 0.61	0.000	1.85 ± 0.53	1.98 ± 0.57	0.011
NLR	2.18 ± 0.97	2.14 ± 0.92	0.589	2.22 ± 0.98	2.16 ± 0.93	0.480
Platelets (10^9^/L)	255.94 ± 60.64	278.32 ± 60.93	0.000	261.38 ± 60.68	274.82 ± 58.72	0.014
MPV (fL)	10.59 ± 1.06	10.15 ± 1.09	0.000	10.57 ± 1.03	10.05 ± 1.06	0.000
PDW (%)	12.56 ± 2.45	12.08 ± 2.19	0.016	12.50 ± 2.16	12.02 ± 2.17	0.016
PCT (%)	0.27 ± 0.05	0.28 ± 0.05	0.001	0.27 ± 0.05	0.27 ± 0.05	0.646
GPC (%)	29.37 ± 8.42	26.91 ± 7.83	0.000	29.73 ± 8.12	26.35 ± 7.53	0.000

*WC* waist circumference;, *BMI* body mass index, *TT* total testosterone, *FT* free testosterone, *FPG* fasting plasma glucose, *FIN* fasting insulin, *HOMA-IR* homoeostasis model assessment-insulin resistance, *CHOL* total cholesterol, *TG* triglycerides, *HDL-c* high-density lipoprotein cholesterol, *LDL-c* low-density lipoprotein cholesterol, *hs-CRP* high-sensitivity, *CRP WBC* white blood cell count, *NLR* neutrophil-to-lymphocyte ratio. *MPV* mean platelet volume, *PDW* platelet distribution width, *PCT* platelet hematocrit, *GPC* great platelet count ratio

### Comparisons between women with PCOS and age-, BMI- and WC-matched women without PCOS

After matching for age, BMI, and WC, women with PCOS exhibited significantly higher levels of TT, FT, FIN, HOMA-IR, TG, and LDL-c compared to the control group (*p* = 0.000, 0.000, 0.009, 0.008, 0.032, and 0.017, respectively). Moreover, the HDL-c levels in the PCOS group were significantly lower than those in the control group (*p* = 0.034). The FPG and CHOL levels did not differ significantly between the two groups (*p* = 0.427, 0.106). Moreover, hs-CRP, WBCs, lymphocytes, platelets, and GPC were significantly higher in PCOS patients compared with the control group (*p* = 0.007, 0.022, 0.011, 0.014, and 0.000, respectively). MPV and PDW were significantly lower in the PCOS group than in the control group (*p* = 0.000, 0.016). However, neutrophils, NLR, and PCT levels did not differ significantly between the two groups (*p* = 0.196, 0.480, 0.646, respectively). (Table 1)

### Decreased MPV was independently associated with the occurrence of PCOS.

Using the occurrence of PCOS as a dependent variable, and age, WC, BMI, TT, FT, FPG, FIN, HOMA-IR, CHOL, TG, HDL-c, LDL-c, hs-CRP, WBCs, neutrophils, lymphocytes, NLR, platelets, MPV, PDW, PCT, and GPC as the independent variables, the binary regression analysis revealed that PCOS development was independently associated with TT, FT, hs-CRP, HDL-c and MPV levels. (Table 2)

**Table 2:** Binary logistic regression analysis of the relationship between MPV and the occurrence of PCOS

	B	S.E.	*P*	Exp(B)(95%CI)
age	−0.109	0.023	0.000	0.896(0.856–0.936)
BMI	0.134	0.030	0.000	1.144(1.079–1.213)
TT	0.055	0.006	0.000	1.057(1.043–1.070)
FT	0.337	0.122	0.006	1.400(1.102–1.779)
hs-CRP	0.114	0.056	0.044	1.120(1.003–1.251)
HDL-c	−1.006	0.378	0.008	0.366(0.174–0.768)
MPV	−0.261	0.093	0.005	0.770(0.641–0.925)
constant	0.107	1.561	1.113	

*BMI,* body mass index, *TT* total testosterone, *FT* free testosterone, *hs-CRP* high-sensitivity CRP, *HDL-c* high-density lipoprotein cholesterol, *MPV* mean platelet volume

### Correlation analysis between MPV and indicators of androgens and glycolipid metabolism

MPV was negatively correlated with BMI, WC, TT, FT, FIN, CHOL, and LDL-c (*r* = −0.101, *p* = 0.005; *r* = −0.108, *p* = 0.002; *r* =−0.177, *p* = 0.000; *r* = −0.124, *p* = 0.001; *r* = −0.072, *p* = 0.045; *r* = −0.086, *p* = 0.016; and *r* = −0.089, *p* = 0.012; respectively). Moreover, MPV was negatively correlated with TT, FT, and CHOL after corrections for age, BMI, and WC (*r* = −0.149, *p* = 0.000; *r* = −0.093, *p* = 0.010; *r* = −0.081, *p* = 0.025; respectively). (Table 3)

**Table 3:** Pearson correlation analysis between MPV and indicators of androgens and glycolipid metabolism

	*r*	*P*	*r*	*P*
age	0.054	0.066	-	-
BMI	−0.101	0.005	-	-
WC	−0.108	0.002	-	-
TT	−0.177	0.000	−0.149	0.000
FT	−0.124	0.001	−0.093	0.010
FPG	0.017	0.643	0.043	0.228
FIN	−0.072	0.045	−0.005	0.890
HOMA-IR	−0.035	0.330	0.029	0.427
CHOL	−0.086	0.016	−0.081	0.025
TG	−0.066	0.067	−0.030	0.402
HDL-c	0.060	0.093	0.021	0.561
LDL-c	−0.089	0.012	−0.069	0.055

*BMI* body mass index, *WC* waist circumference, *TT* total testosterone, *FT* free testosterone, *FPG* fasting plasma glucose, *FIN* fasting insulin, *HOMA-IR* homoeostasis model assessment-insulin resistance, *CHOL* total cholesterol, *TG* triglycerides, *HDL-c* high-density lipoprotein cholesterol, *LDL-c* low-density lipoprotein cholesterol

## Discussion

Many recent studies demonstrate that PCOS represents a chronic low-grade inflammatory condition [9, 10], with evidence suggesting this inflammatory state contributes to both ovarian dysfunction and metabolic disturbances in affected patients [11]. Given the central role of chronic inflammation in PCOS, investigating different inflammatory markers has gained importance. One study demonstrated that serum calprotectin levels were significantly elevated in PCOS patients and were positively correlated with IR, WC, and TT levels [12]. Similarly, telomerase activity was found to be reduced in PCOS patients, with negative correlations observed between telomerase levels and metabolic syndrome (MS) markers, LDL-c, and TG [13]. These findings suggest that inflammation in PCOS may influence cellular aging processes and metabolic complications. Additionally, the prevalence of temporomandibular joint disorder (TJD) was significantly higher in PCOS patients, and this condition was particularly associated with reduced progesterone levels and increased TNF-α, MMP-1, and MMP-8 levels [14]. These results highlight the broad systemic effects of PCOS and further support the crucial role of inflammation in its pathogenesis.

Classically, inflammation is an adaptive response triggered by noxious stimuli or conditions, like infection and tissue damage, which causes leukocyte and plasma protein recruitment to the affected site [15]. In contrast to classical inflammation, PCOS is considered to be in a state of chronic low-grade inflammation (also called para-inflammation), which is an immune state between basal homeostasis and the classical inflammatory response, an adaptive response induced by tissue stress or dysfunction, and is largely dependent on tissue macrophages [15].

Neutrophils and lymphocytes are the two most abundant leukocyte subtypes that are of greatest concern in PCOS patients. Neutrophils primarily reflect systemic inflammatory status, while lymphocytes serve as indicators of cellular immune responses [16]. In recent years, several studies have analyzed changes in neutrophils, lymphocytes, and NLR in PCOS patients. A 2022 meta-analysis of 13 clinical studies showed that PCOS patients displayed enhanced NLR compared to controls due to increased leukocyte and neutrophil levels as well as a reduction in lymphocytes [16]. Compared to our controls, the leukocytes and neutrophils were significantly increased in PCOS patients; however, the lymphocytes were significantly increased despite no reduction, and NLR was not significantly different between the two groups. This finding aligns with multiple observational studies conducted in Chinese populations [9, 17–19], including one with a sample exceeding 2,000 cases [17]. Xiong et al. proposed that lymphocytosis could contribute to chronic inflammation and hormonal dysregulation in PCOS patients [9]. Although some of the above studies omitted specific NLR values, the NLR was not significantly increased in the PCOS group based on the values of neutrophils and lymphocytes [9, 17, 18]. The finding that lymphocytes are elevated in patients with PCOS is not surprising, as previous studies have reported a positive correlation between the occurrence of hyperandrogenism, IR, and MS and lymphocyte counts [20, 21]. In addition, sample size and genetic background may be partly responsible for the difference in results. Notably, 9 of the 13 studies analyzed in the meta-analysis originated from Turkey, and approximately half involved fewer than 100 participants [16]. Discrepancies persist even among studies examining similar genetic backgrounds [22–24]. Larger, multicenter studies with diverse genetic profiles are required to clarify the alterations in peripheral blood lymphocyte counts and NLR observed in PCOS patients.

Platelets are not only involved in hemostasis but also in the inflammatory responses that occur in natural organisms. Emerging evidence demonstrates that platelet count and MPV effectively differentiate inflammatory conditions and correlate with disease severity.

Platelet counts were significantly higher in PCOS patients than in controls, even after adjusting for age and obesity. While previous studies have reported inconsistent findings regarding platelet count changes in PCOS, most evidence suggests these patients exhibit elevated platelet counts, whether directly or indirectly [6, 7, 25]. Additionally, elevated platelet counts in patients with PCOS may result from heightened pro-inflammatory cytokine activity or dyslipidemia. A study by Korniluk et al. showed that pro-inflammatory cytokines, especially interleukin-6, promote platelet formation by either enhancing the liver’s thrombopoietin production or acting on megakaryocytes via membrane receptors directly [26]. Ferroni et al. further established that dyslipidemia induces excessive platelet factor secretion, which mobilizes bone marrow progenitor cells and ultimately promotes thrombocytosis [27].

In our study, MPV was significantly decreased in PCOS patients regardless of age and obesity corrections. Another Saudi Arabian study on the PCOS population was consistent with our results [28]. Additionally, some previous large-sample studies provide significant support for this result from different perspectives. Park et al. reported that platelet counts were significantly higher while MPV was significantly lower in women with MS compared to those without it. However, both of them exerted independent influences on the development of MS in women [7]. Hou et al. found that patients with higher obesity indices (BMI, WC, or waist-to-hip ratio) were at a greater risk of developing lower MPV and PDW; however, both of them reduced progressively as the obesity index increased [29]. Moreover, PCOS patients are at a significantly increased risk of developing MS and obesity. However, a 2022 meta-analysis reported significantly higher MPV levels in PCOS patients than in controls [5]. This discrepancy may stem from genetic variations and Limited sample sizes, as 9 of the 10 analyzed studies involved Turkish populations with small cohorts [5]. Further multi-ethnic studies with larger samples should clarify the association between MPV alterations and PCOS.

Although the relationship between MPV changes and PCOS pathophysiology remains unclear [2]. Prior studies have proposed the following possible explanations for the significant reduction in MPV in inflammatory diseases: ①Increased small-volume platelet production: Overproduction of pro-inflammatory cytokines and acute-phase reactants reduces MPV by impairing megakaryopoiesis and releasing a small volume of platelets from the bone marrow [30, 31]. ②Increased platelet count: In a more physiological state, MPV is inversely proportional to platelet count, and PCT remains constant [7, 32]. As the inverse relationship between MPV and platelet count might be disrupted by the worsening of the inflammatory state [26]. In the present study, we observed no significant difference in PCT between the two groups after matching for age and obesity. Therefore, the state of our study population might be more biased toward basal homeostasis. ③Large inflammation-induced platelets are either heavily utilized or sequestered by the inflammation site, resulting in a reduction of circulating MPV [32, 33]. In the present study, the decrease in MPV in PCOS patients may be due to a combination of increased platelet counts and massive depletion of large platelets at the site of inflammation, because platelet counts were increased but large platelet counts were significantly decreased in PCOS patients in this study population. Future investigations should clarify MPV dynamics in Chinese PCOS populations and elucidate the underlying molecular pathways.

In our study, MPV maintained a significant negative correlation with TT, FT, and CHOL levels after adjusting for age and obesity. Nonetheless, the correlation between MPV and CHOL is not unusual because dyslipidemia causes increased platelet counts [27, 34]. Regarding the correlation between MPV and androgens, a study by Dogan et al. et al. showed a significant negative correlation between MPV and TT and DHEAS levels in patients with PCOS [35], which supports the findings of this study. However, there are also studies that hold a different opinion, suggesting a positive correlation between MPV and FT levels in PCOS patients [36]. The precise relationship between MPV and both hyperandrogenism and dyslipidemia remains unclear, warranting further investigation.

Despite these findings, our study has several limitations. First, the participants were exclusively recruited from Xuzhou Central Hospital and restricted to those with complete medical records, which may limit the generalizability of our results to all Chinese women with PCOS. While this sampling approach does not invalidate our primary conclusions, multicenter studies with larger cohorts are required for validation. Additionally, this study was a retrospective case-control study, therefore, we were unable to assess the causal relationship between changes in various inflammatory markers and the onset and progression of PCOS. Future prospective studies should address this limitation. Finally, HOMA-IR provides only an approximate measure of IR in patients.

In summary, Chinese PCOS patients have significantly reduced MPV as an inflammatory indicator independent of obesity, IR, and dyslipidemia.

## Data Availability

The original contributions presented in the study are included in the article, further inquiries can be directed to the corresponding author.
